# Floccular fossa size is not a reliable proxy of ecology and behaviour in vertebrates

**DOI:** 10.1038/s41598-017-01981-0

**Published:** 2017-05-17

**Authors:** S. Ferreira-Cardoso, R. Araújo, N. E. Martins, G. G. Martins, S. Walsh, R. M. S. Martins, N. Kardjilov, I. Manke, A. Hilger, R. Castanhinha

**Affiliations:** 1 Institut des Sciences de l’Évolution – Université de Montpellier, Place Eugène Bataillon, 34095 Montpellier cedex 05, France; 2 GEAL - Museu da Lourinhã, Rua João Luís de Moura 95, 2530-158 Lourinhã, Portugal; 3 LATR/IST/CTN - Campus Tecnológico e Nuclear, Estrada Nacional 10 (km 139.7), 2695-066 Bobadela, LRS Portugal; 4 Instituto de Plasmas e Fusão Nuclear, Instituto Superior Técnico, Universidade de Lisboa, Av. Rovisco Pais 1, 1049-001 Lisboa, Portugal; 5 Museum für Naturkunde - Leibniz-Institut für Evolutions- und Biodiversitätsforschung, Invalidenstraße 43, 10115 Berlin, Germany; 6 Huffington Department of Earth Sciences, SMU, 75275-0395 Dallas, Texas USA; 7 Instituto Gulbenkian de Ciência – Rua da Quinta Grande 6, 2780-156 Oeiras, Portugal; 8 CNRS-UPR9022 - Institut de Biologie Moléculaire et Cellulaire, 15 Rue René Descartes, 67084 Strasbourg, Cedex France; 9 cE3c – Centre for Ecology, Evolution and Environmental Changes, Faculdade de Ciências, Universidade de Lisboa, Campo Grande, 1749-016 Lisboa, Portugal; 10 Department of Natural Sciences, National Museums Scotland, Chambers Street, EH1 1JF Edinburgh, UK; 11 CENIMAT/I3N, Faculdade de Ciências e Tecnologia, Universidade Nova de Lisboa, Quinta da Torre, 2829-516 Caparica, Portugal; 12 Helmholtz Centre Berlin for Materials and Energy (HZB), Institute of Applied Materials, Hahn-Meitner-Platz 1, 14109 Berlin, Germany

## Abstract

The cerebellar floccular and parafloccular lobes are housed in fossae of the periotic region of the skull of different vertebrates. Experimental evidence indicates that the lobes integrate visual and vestibular information and control the vestibulo-ocular reflex, vestibulo-collic reflex, smooth pursuit and gaze holding. Multiple paleoneuroanatomy studies have deduced the behaviour of fossil vertebrates by measuring the floccular fossae (FF). These studies assumed that there are correlations between FF volume and behaviour. However, these assumptions have not been fully tested. Here, we used micro-CT scans of extant mammals (47 species) and birds (59 species) to test six possible morphological-functional associations between FF volume and ecological/behavioural traits of extant animals. Behaviour and ecology do not explain FF volume variability in four out of six variables tested. Two variables with significant results require further empirical testing. Cerebellum plasticity may explain the lack of statistical evidence for the hypotheses tested. Therefore, variation in FF volume seems to be better explained by a combination of factors such as anatomical and phylogenetic evolutionary constraints, and further empirical testing is required.

## Introduction

Valuable insights into the biology of extinct animals can be gained through comparison of fossil remains to the osteology of extant taxa. The skull has been widely used to investigate the sensory context of ancient life forms and, with the advent of computed tomography, to gain access to endocranial neuroanatomical data for interpretation of the brain architecture of extinct species^1–3^. However, the relationship between the behavioural and ecological implications of brain morphology remains mostly speculative.

The floccular fossa lobes of the cerebellum are a centre for integration of visual and vestibular stimuli and control of the extraocular muscles^4, 5^. Floccular fossae (FF) are present in distinct groups of animals such as: dinosaurs (birds, non-avian theropods, ornithopods and sauropods)^6–9^, pterosaurs^1^, early synapsids^2, 10, 11^ and mammals^10, 12^. In extant animals (mammals and birds) the vestibulocerebellum can be directly analysed and is composed of the flocculus, paraflocculus, nodulus and ventral uvula^13–15^. The function of a cerebellar lobule is determined by its connections^16–19^. The floccular and parafloccular lobules regulate compensatory movement of the eyes to respond to rotational movements of the head (vestibulo-ocular reflex, VOR) or to track a moving object in the field of view (smooth pursuit), but also contribute to stabilize the head via cervical muscles (vestibulo-collic reflex, VCR)^5, 14, 20^. However, eye movement is also controlled by other cerebellar tissues. For example, the VOR may be compensated after lesions^21–23^, showing that the cerebellum possesses redundant or adaptable neuronal structures capable of compensating for particular functional deficits. The VOR has been suggested to be particularly important during aerial and aquatic rapid manoeuvres or highly active predatory activity^1, 8, 24^. For instance, elite gymnasts show enhanced VOR and VOR cancellation gains during aerial manoeuvres, which enhances visualization of the landing area^25, 26^. The dorsal paraflocculus of some mammals is involved in saccade and smooth pursuit regulation, but its connections suggest these structures may also be involved in arm movement control, with focus on visually guided reaching^27, 28^. Therefore, there are only very limited studies presenting physiological data for an oculomotor function of the dorsal paraflocculus and any other functions are still largely unknown. Given the putative correlation between the vestibulocerebellum volume and animal locomotion, the variation of cerebellar flocculus and paraflocculus volumes have been previously investigated^1, 10^. In this context, the exact functions of the paraflocculus and flocculus have been extremely elusive to identify, given that there are very little empirical evidence supporting any particular functional hypothesis. Therefore, to our knowledge, the implications of FF size for deducing behaviour have not been widely tested across amniote groups.

Anatomically, we here define the Floccular Fossae Lobe (FFL) as the neural tissue that fills the antero-medial fossa present in the periotic bone complex of some vertebrates (petrosal of mammals; opisthotic + prootic in birds). We opted to use the term Floccular Fossae (FF) only when referring to the fossae in the periotic bones and FFL only when referring to the neural tissue that projects into the FF. Thus, the FFL includes the flocculus and paraflocculus in birds^13^. In mammals only the paraflocculus (petrosal lobe) forms the FFL, but some mammals have different parafloccular contributions to the FFL^16^. For example, in monkeys, the portion of the cerebellum that is housed by the floccular fossa is composed of the petrosal lobule (part of the dorsal paraflocculus), while in other mammals it is usually formed by the entire paraflocculus, where it is also known under the same name (petrosal lobule)^5, 16^. Thus, it is important to note that although the floccular fossa may have the same name in birds and mammals, it may house different structures in different animals. In addition, due to lack of functional experiments across a wide range of taxa, even when the FF house anatomically similar structures, the homology of their function is usually uncertain.

Despite all of this uncertainty, multiple paleontological studies have been conducted based on a double assumption, namely that: a) brain endocasts are a good proxy for *in vivo* brain volume^12, 29–34^ and b) large FFL volumes present in animals that occupy a wide range of habitats (e.g. arboreal, aerial and even aquatic and terrestrial) result from adaptive pressures to produce lighter, faster or more manoeuvrable animals^1, 6, 8, 10, 11, 35^ due to shared selective pressures to develop sharply coordinated movements^1, 4, 6, 11, 12, 30, 32, 33, 35–39^. However, the reliability of FF dimensions as a means of providing insight into the behaviour of extinct amniotes has never been adequately tested^18^. It is crucial to collect experimental data regarding FF sizes from multiple extant taxa (where behaviour can also be directly observed), to test previous speculative conclusions. Thus, only by using direct measurements can we test if what has been previously proposed in the literature, regarding the morpho-functional correlations of the vestibullocerebellum, has any empirical support.

We used X-ray computed tomography scans (CT-scans) to render digital braincase endocast models of the brain cavity of a large sample of extant mammals and birds with a wide range of behaviours. Floccular fossae volumes (as a proxy for the neural tissue that forms the FFL) were used to investigate possible correlations between FFL size and: H1) body mass; H2) agility; H3) 2D/3D locomotion; H4) locomotor type; H5) feeding; H6) activity pattern.

Phylogenetic generalized least-squares (PGLS) was used to obtain FF relative values (residuals) and to run multivariate analyses. Our results suggest that the cerebellum is a functionally plastic structure easily adaptable, that hinders the establishment of direct causal relationships between morphology and function.

## Results

We gathered the largest dataset so far created for neuroanatomical comparisons of cerebellar volumes (n = 59 for birds and n = 48 for mammals). In general, we find little to no correlation between behaviour or ecology and relative FF size (see Table 1). Variability within categories is relatively high, both in mammals and birds, and body mass distribution presents no correlation with FF size. The results here presented were obtained with the best fitting models (see Supplementary Materials and Methods). For descriptive statistics of collected data see Supplementary information III.

**Table 1:** Hypotheses formulated to test the relationship between FF size and ecology/behaviour.

	Mammals	Birds
H I (Body mass)	Not correlated	Not correlated
H II (Agility)	Not correlated	*
H III (2D/3D locomotion)	Not correlated	Not correlated
H IV (Locomotor type)	Not correlated	*
H V (Feeding)	Not correlated	Verified
H VI (Activity pattern)	Not correlated	Verified

*Tested in Walsh *et al*.^8^.

### Mammals

The taxon with the largest FF (absolute volume) analysed here is *Lagotrix lagotricha* (Humboldt’s woolly monkey) with 560.99 mm^3^, and the taxon with the smallest FF volume is *Mus musculus* (house mouse) with 3.81 mm^3^. The largest relative FF volume is present in *Talpa europaea* (European mole) with a FF volume that occupies 2.34% of the total brain endocast and the smallest relative FF volume belongs to *Vulpes vulpes* (red fox) at 0.04% (see Supplementary information I and II for further analysis and results).

**Figure 1:**
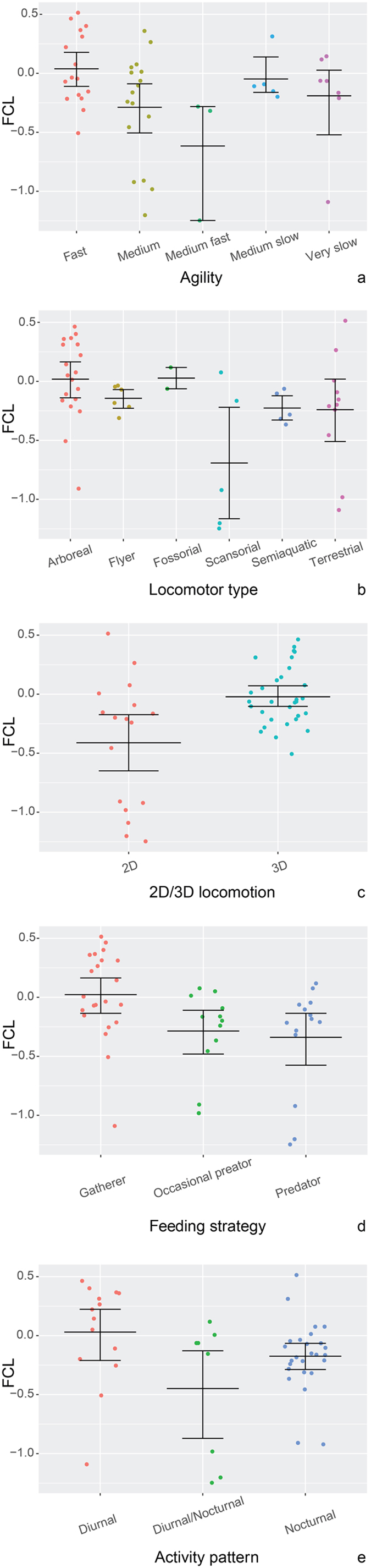
Dotplots of mammal FF relative size grouped according to agility (**a**), locomotor type (**b**), locomotion dimension (2D/3D) (**c**), feeding strategy (**d**) and activity pattern (**e**). Jittered X axis values. n = 48, mean for the pooled data = −0.160, s.e.m. ± 0.060. Mean and error bars (standard error of mean) are shown for each group. For descriptive statistics of each category see Supplementary information I.

There is no significant correlation between FF relative size and body mass (see Table 2). Agility categories do not separate species according to FF relative sizes (see Table 2). The FF relative size does not vary with 2D/3D locomotion and locomotor type (see Table 2). The results remain unaltered when the “fossorial” category (which had only two specimens) is removed from the analysis. The analysis also did not reveal differences between activity pattern and feeding categories (see Table 2). Removal of the “Diurnal/Nocturnal” category does not change the results. The analysis of dotplots reveals considerable variability within each ecological niche (see Fig. 1 and Table 2 for more details). We calculated the phylogenetic signal of FF volume and found a significant tendency for closely allied species to resemble each other (Pagel’s λ = 0.93).

**Table 2:** Results of the analyses of variance of the mammal dataset (Brownian Motion).

	Statistics	
	All variables	Stepwise removal
Body mass	Chisq = 0.78; df = 1; p = 0.38	Removed
Agility	Chisq = 2.28; df = 4; p = 0.68	Removed
Locomotor type	Chisq = 3.63; df = 5; p = 0.60	Removed
Locomotion dimension (2D/3D)	Chisq = 1.44; df = 1; p = 0.23	Removed
Feeding	Chisq = 1.85; df = 2; p = 0.40	Removed
Activity pattern	Chisq = 2.68; df = 2; p = 0.26	Removed

Statistics of the effect of each predictor on FF relative size variation (Chi-square value, degrees of freedom and p value; α = 0.05).

### Birds

**Figure 2:**
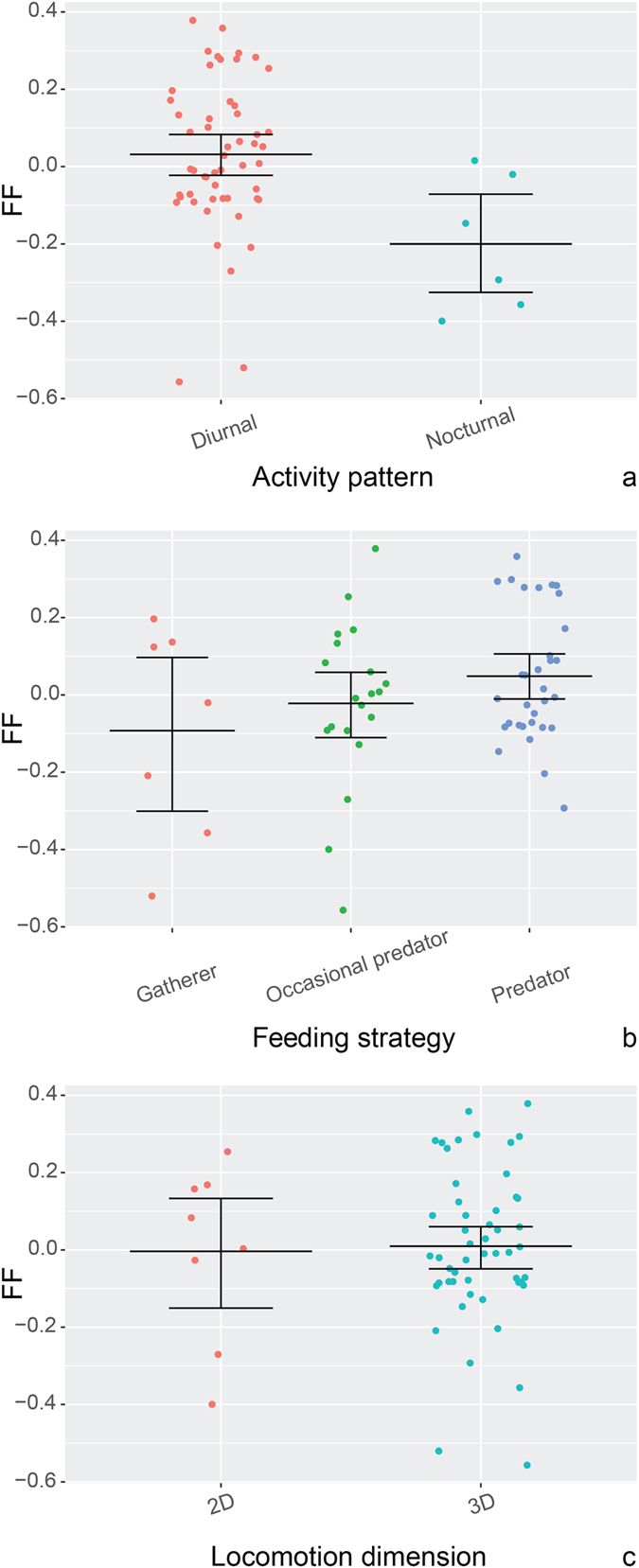
Dotplots of bird FF relative size grouped according to activity pattern (**a**) and feeding strategy (**b**). Jittered X axis values. n = 59, mean for the pooled data = 0.002, s.e.m. ± 0.026. Mean and error bars (standard error of mean) are shown for each group. For descriptive statistics of each category see Supplementary information II.

Our analysis of the bird data also does not reveal a significant correlation between FF relative size and body mass (see Table 3). However, the analysis of variance reveals a difference in average FF relative size of nocturnal vs. diurnal birds, with the latter group having a larger FF relative size (see Table 3). There are differences among feeding categories, with predators exhibiting the highest values on average (see Table 3). All categories show high variability (Fig. 2), with gatherers and occasional predators exhibiting a wide range of values (see Fig. 2).

**Table 3:** Results of the analyses of variance of the bird dataset (Brownian Motion).

	Statistics	
	All variables	Stepwise removal
Body mass	Chisq = 2.27; df = 1; p = 0.13	Chisq = 2.33; df = 1; p = 0.13
2D/3D Locomotion	Chisq = 0.01; df = 1; p = 0.93	Removed
Feeding	Chisq = 10.92; df = 2; p = **0.00(4)**	Chisq = 11.59; df = 2; p = **0.00(3)**
Activity pattern	Chisq = 10.99; df = 1; p = **0.00(0)**	Chisq = 11.31; df = 1; p = **0.00(0)**

Statistics of the effect of each predictor on FF relative size variation (Chi-square value, degrees of freedom and p value; α = 0.05). Standard error of the regression: 0.03.

Overall, FF relative size differs between feeding and activity pattern categories in birds. Note that this result contrasts with that from mammals, where we do not find significant correlations for any of the ecological categories tested. However, significant values in birds can result from the introduction of the activity patterns category in the multiple analysis, which includes a group (nocturnal) with only six specimens. The analysis retrieves no significant results when this variable is removed. When testing each variable independently the differences are smaller between groups, since feeding strategy was not related to FF volume (p = 0.09). In both mammals and birds, FF relative size does not correlate with body mass and the values within ecological categories demonstrate high variability (Figs 1 and 2).

## Discussion

In mammals, FF relative volume does not correlate with any of the tested biological variables. Diurnal birds seem to exhibit higher FF relative volumes than nocturnal birds, but other tested variables do not have a consistent effect on FF volumetric variation.

Our results oppose widespread ideas about the causes of volumetric variation of the FF in extant vertebrates^1, 6, 8, 10, 11, 35, 40–42^. Gannon and colleagues have suggested that body size might explain FF volume variation in mammals^12^. Because body mass influences animal locomotor behaviour, we expected to obtain a negative trend in our sample. However, body mass, locomotor type, 2D/3D locomotion and agility revealed no correlation with FF size. These results agree with previous conclusions regarding birds^8^ and confirm a similar trend in mammals.

The avian plesiomorphic condition present in *Archaeopteryx* ^43^ and many bird-like non-avian theropod dinosaurs seems to be enlarged FF volumes^18, 37^. Consequently, it is possible that the large FF of extant birds is a retention of this ancestral condition. If so, this might explain the absence of statistical correlations between FF size and body mass or agility in birds^5, 8^.

Although the presence of large FF in extant mammals could also relate to phylogeny^44^, the variability is much higher, ranging from complete absence of fossae to deep depressions in the periotic complex^12, 33, 45^. Even within groups, such as primates, we found the FF may be absent or present.

Walsh *et al*.^8^ suggested that flightless birds might have maintained relatively large FFL volumes as a result of exaptation from flight-related use, to functions related to running and swimming. A similar exaptation is less easy to envisage in some mammals, as in *Talpa europaea* (European mole), which possesses a relatively large FF volume, comparable to those of gliding or arboreal species (e.g., *Petaurus* sp. the flying phalanger or *Cebus apella* the tufted capuchin) despite being practically blind, not particularly agile, and not being directly descended from a gliding or arboreal ancestor. Since we found no relationship between FF size and two- or three-dimensional locomotion, FF size in mammals does not seem to be directly influenced by three-dimensional locomotion, unlike in flightless aquatic birds, which require VOR processing for swimming in three dimensions.

Surprisingly, echolocating and non-echolocating bats do not form two clusters when FF relative size is analysed. Since echolocating bats do not use vision for navigation, one would expect their FF relative volumes to be significantly smaller than those of visual bats. It has been previously shown that the cerebellum presents a high degree of functional plasticity^21, 23^, and it is possible that, if required, parts of the flocculus involved in occular motor control could adapt to new functions. Consequently, an exaptation of floccular neural tissue for functions other than eye movement control, or changes in occupancy of the FF by parts of the paraflocculus engaged in other functions (e.g. dorsal paraflocculus), might explain why both mammals and birds exhibit large FF volumes within a wide range of ecological and behavioural contexts.

It seems possible that the FFLs could also be involved in processing vestibular cues in blind animals. For instance, where vision has been partially or totally lost in some taxa, vestibular cues become more relevant for navigation^46^. The loss of visual cues can be compensated by an increased relevance on other cues during an individual’s lifetime^47^, so it is not surprising that the same may happen during macroevolutionary processes. Large FFLs might be advantageous if directional selective pressures are present, particularly in low light conditions. In this case, the neural tissue that forms the FFLs and that is typically involved in processing visual cues would be free to be co-opted to process other types of stimuli. Given the importance of vestibular cues and proprioception for balance, the potential for readjustment of FFLs functions in certain species should not be disregarded. To our knowledge, this possibility has not been tested.

Also unexpected was the lack of correlation between feeding strategy and activity pattern with FF size in mammals. These two variables served as a control to body mass, agility and locomotor type, because behaviour and biomechanics are correlated with the type of resources exploited by the animals^48^. This may be explained by ecological guilds being composed of animals that differ in shape, size and behaviour^49^. Consequently, even animals exploiting the same resource could differ in how that resource is used as a result of differences in microhabitat occupation or slight food preference^49^. In our dataset, although species can be grouped into very general guilds, they differ in many behavioural aspects (e.g., although *Tyrannus tyrannus* and *Hirundo rustica* are both fly-catcher species, their behavioural aspects and biomechanical constraints are clearly distinct).

Regarding activity patterns, the absence of correlation between FF size variation and available light can be explained by the evolutionary history of mammals. Mammals evolved as nocturnal animals, and their vision remains in general equally adapted to both diurnal and nocturnal life styles^50, 51^. Furthermore, strict diurnality is present in very limited mammalian clades^52, 53^, given that many mammals are active both during day and night (cathemeral).

Unlike mammals, the difference of FF size between feeding and activity pattern categories is significant in birds (see Table 3). Although the variability is high, predators have relatively larger FFs than occasional predators, and the group with the smallest relative FF sizes is the gatherer group. This is consistent with the importance of visual accuracy in animals that rely heavily on sight to locate, identify and pursue prey. These results support the hypothesis that theropod dinosaurs’ cerebellar growth (including FFLs) is related to acquisition of pursuit predatory habits^6^. The presence of larger FF in non-avian theropods may thus indicate an adaptation to active pursuit predation that was extremely useful during flight acquisition.

According to the data presented here, nocturnal birds show significantly smaller FF relative size than diurnal species (p < 0.01). This result provides further support to the idea that nocturnal birds are not fully dependent on vision to hunt prey. For instance, barn owls are capable of locating their prey in total darkness using only auditory cues^54–56^, and the cave dwelling oilbirds that live in low light environments^57^ possess eyes that are extremely sensitive to light, but also rely on echolocation and tactile cues^58, 59^. Therefore, importance of vision in object identification and muscular control of the eye might become less relevant in these nocturnal taxa. Alternatively, it may be suggested that given that barn owls have exceptionally large telencephala (especially the telencephalic region known as the Wulst)^29, 60^ the relative volume of FFLs might have been reduced.

Univariate analysis retrieved a non-significant result for feeding. These apparently contradictory results indicate the importance of including several independent behavioural parameters to identify relevant eco-morphological correlations. In addition, it is important to note the small number of nocturnal specimens (n = 6) in the analysed sample, as well as the variability presented by all three feeding categories. Thus, before extracting any definitive conclusions regarding the effect of these variables in birds, further investigation is required with a broader sample of nocturnal birds.

Although specific cerebellar folia are responsible for different functions^5^, interactions between cerebellar structures are complex and difficult to model. Additionally, there is little knowledge about the function of paraflocculi in extant mammals and birds. Thus, strong evidence is required before structural-functional correlations can be established. Optimal ocular-motor coordination involves several cerebellar components, and a reductionist approach to the region involving isolation of individual structures for linkage to specific functions is problematic^23, 61–64^. Moreover, significant VOR gain variation has been registered in individuals of the same species (e.g., elite gymnasts vs. amateur gymnasts)^47^, suggesting that interspecific functional variation could be unpredictable and difficult to interpret. The apparent absence of a correlation between FF size and ecology/behaviour may be also explained by a tradeoff between the limits of the functional plasticity of cerebellum and FFLs^21, 27, 28^, and constraints resulting from particular cranial architectures^10^. Moles, echolocating bats and cetaceans possess large FFLs^45, 65^, yet none of these groups rely primarily on vision. Paulin^45^ suggested that this part of the cerebellum could be involved with processing echolocation in bats and cetaceans but we find no evidence for this possibility. Witmer and colleagues^1^ suggested that the membranous wings of pterosaurs may have projected proprioceptive fibers to the central nervous system, and that this might explain the unusually large FFL in this group of flying archosaurs. For this to have occurred in pterosaurs, the flocculi of these animals must have undergone relatively extreme adaptations to process impulses arriving from proprioceptive afferents, as relatively little proprioceptive processing is known to occur in this structure in modern animals^66^.

However, in at least some extant flying animals with extensive membranous wings (bats) the FFLs may not control image stabilization in the retina, particularly in echolocating bats. In this case, the FFLs function is likely related to integration of vestibular input, given the vestibulo-cerebellar tract connection between FFLs and semicircular canal system, or to limb movement control^28^. Nevertheless, it is also possible that pterosaurs, having no exact modern analogue, possessed neurosensory capabilities not seen in living taxa. Consequently, the purpose of the enlarged pterosaur FFL remains speculative^8^.

Although cetacean FFLs are less pronounced than in other mammals, their FF values are still relatively large^67, 68^. The relationship between the cerebellum as a whole and the periotic and prootic bones is key to understanding any evasive cranial architectural constraints to FF dimensions that might be acting. The size of FF and FFL may depend on the orientation, position and development of these bones as a consequence of the enlargement of the cerebellar hemispheres. Olson^10^ suggests that, in sagittal view, the periotic gradually rotates from a vertical (more basal) to a horizontal (more derived) position and that this is correlated with an increased cerebellar hemisphere size in mammals. At first, this seems to be independent of FF size. Nevertheless, the relative position of the periotic may be an important factor. If the periotic is more medially positioned, the physical pressure of the FFL growth during development could require (or even produce) a deeper FF due to lack of space to house the hemispheric portion of the cerebellum. Tracing the evolution of the periotic with respect to the cranial wall could help to clarify how this bone was modified as the cerebellar hemispheres enlarged. Walsh and colleagues^8^ argued that the FFL volume increase in birds could be a consequence of an enlargement of other parts of the cerebellum rather than FFL tissue itself. Protrusion of FFLs into the periotic/prootic could be related to an increase in uvulo-nodular tissue within the cerebellum^8^, or even to a combination of this and constraints imposed by cranial architecture.

In summary, our data do not support FF size as a reliable proxy for inferring ecology and behaviour within the synapsid and diapsid lineages in most cases. The FF size is probably more affected by adaptive (e.g. co-option to process navigation and spatial perception in blind animals) or even non-adaptive (e.g. phylogenetic or anatomical constraints) factors. Currently, the limited knowledge about the paraflocculi functions and processing capacities precludes any definitive conclusions. Thus, more experimental work needs to be done across multiple taxa, and particularly after implementation of alternative protocols in the study of the cerebellum^69^.

Thus, with the present knowledge, and until further empirical testing can be conducted, inferences of relationships between FF size and ecological (or behavioural) traits in extinct animals should be treated with caution.

## Materials and Methods

A total of 47 extant mammal species and one Anomodontia species were selected to cover the widest ecological range possible (see Supplementary Materials and Methods). From these, 27 skull specimens from the mammal collections of the Museum für Naturkunde (MfN) were scanned at the Helmholtz Zentrum Berlin (HZB).

Our avian dataset (59 species of extant birds) corresponded to the values for the FF cast volume and endocast volume published by Walsh *et al*.^8^.

**Figure 3:**
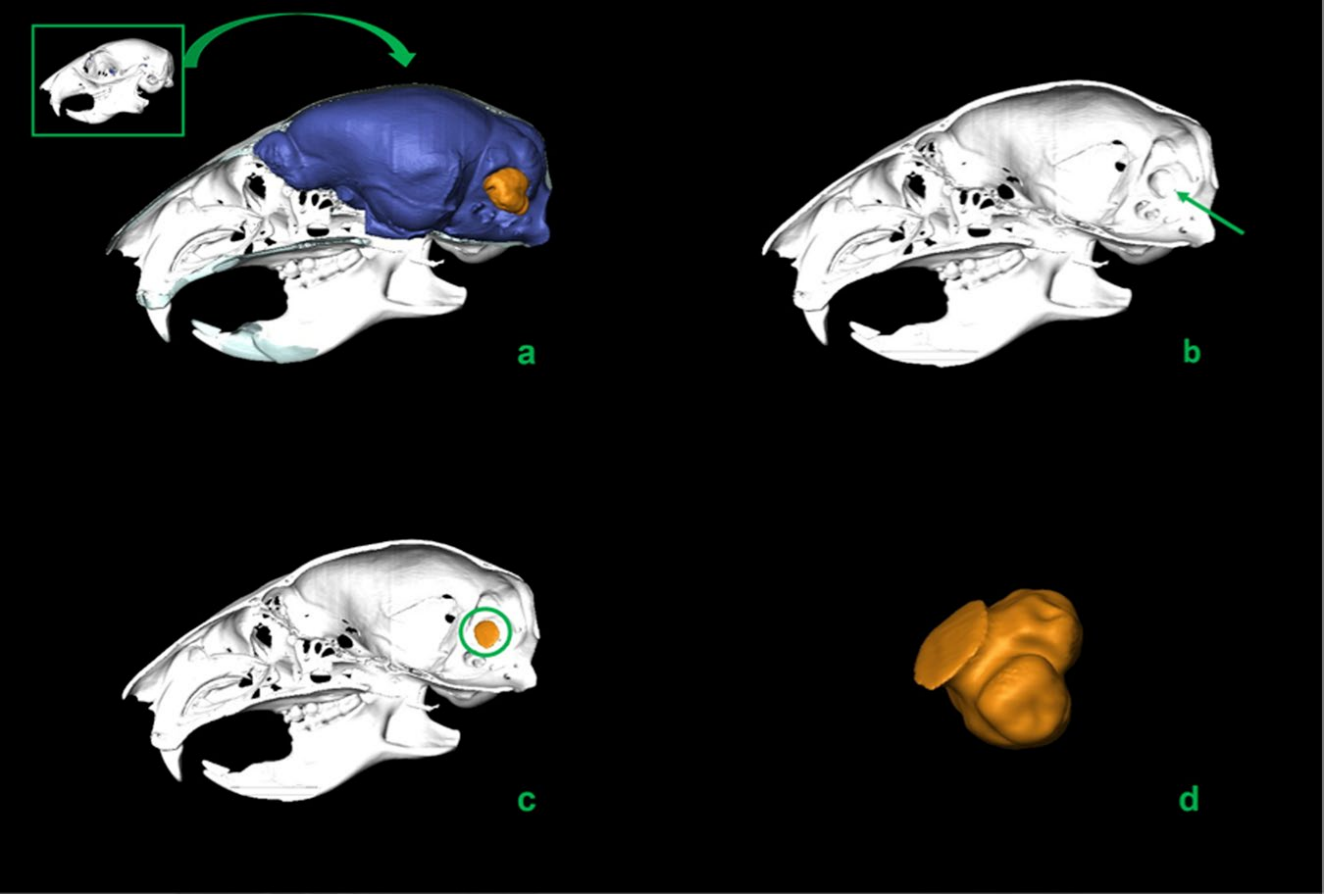
Segmentation process of FF volume: lateral view of a *Sciurus vulgaris* (red squirrel) skull before (top left corner) and after removing the left half of the skull (**a**); left lateral view with indication of the FF (green arrow) (**b**); volume of the right FF selected (green circle) (**c**); right FF volume in posterior view (**d**).

**Figure 4:**
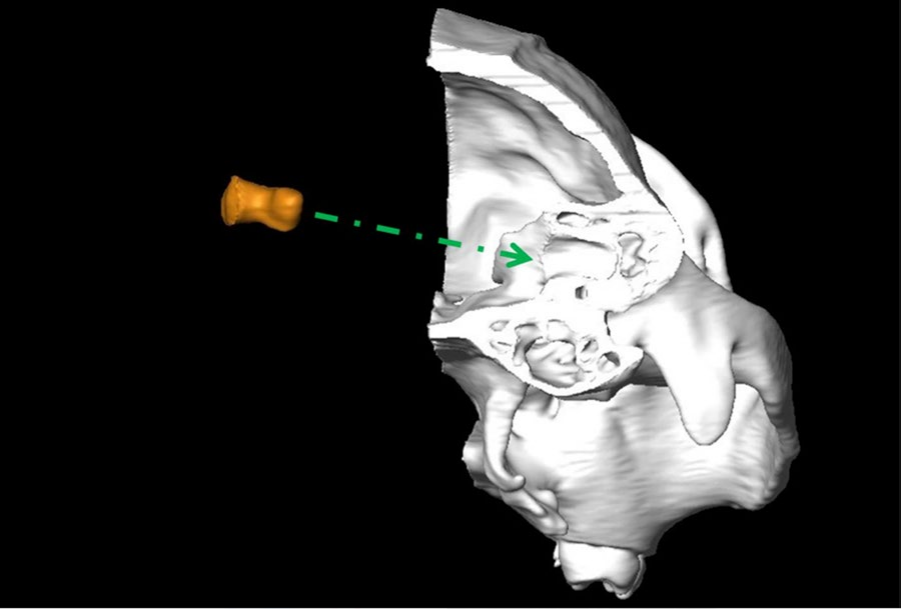
Coronal cut-out of the 3D reconstruction of the right half of *Alouatta caraya* skull (in posterior view (left side removed). FF endocast in orange.

**Figure 5:**
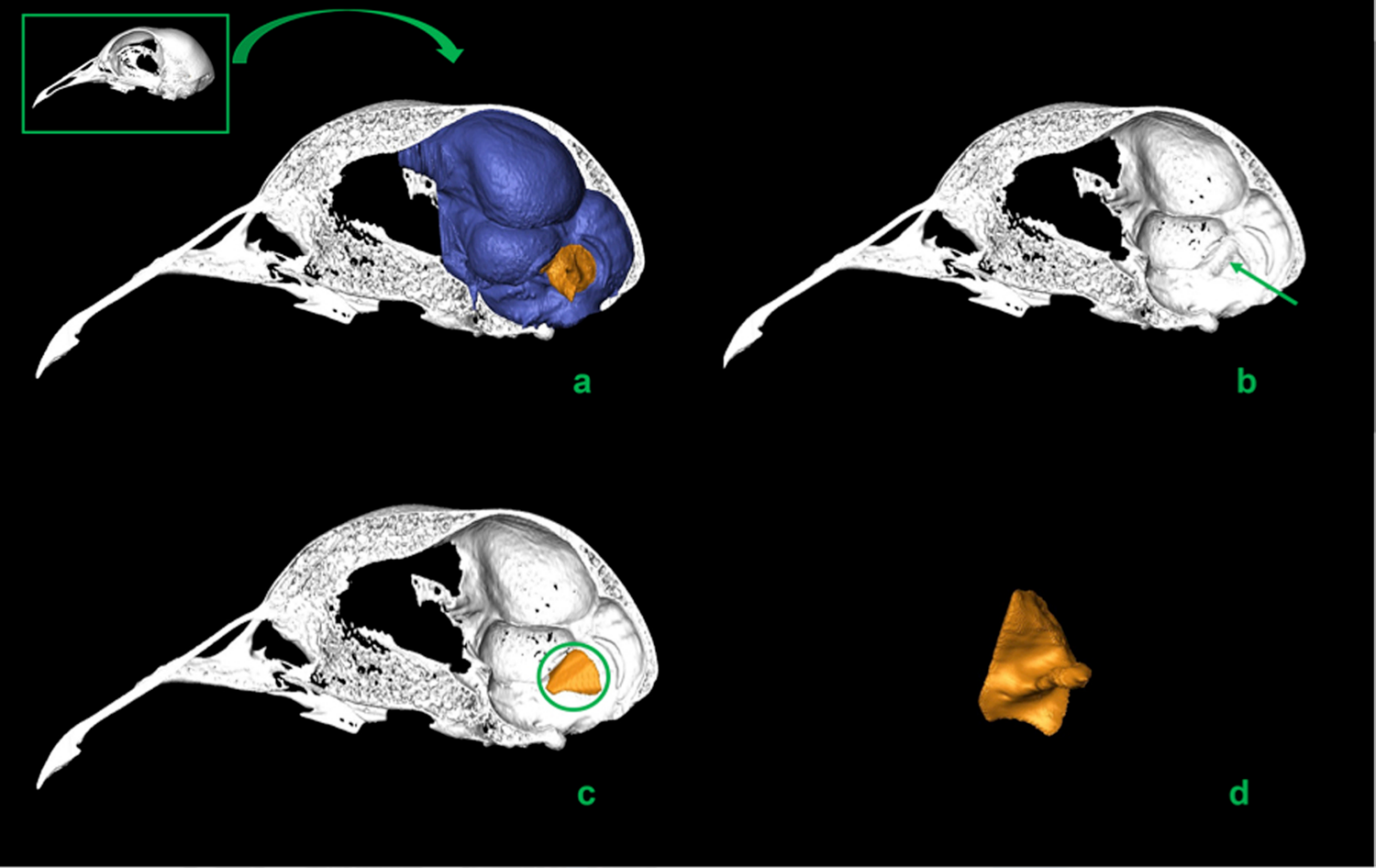
Segmentation process of FF volume: lateral view of a *Columba livia* skull before (top left corner) and after removing the left half of the skull (**a**); left lateral view with indication of the FF (green arrow) (**b**); volume of the right FF selected (green circle) (**c**); right FF volume in postero-lateral view (**d**).

**Figure 6:**
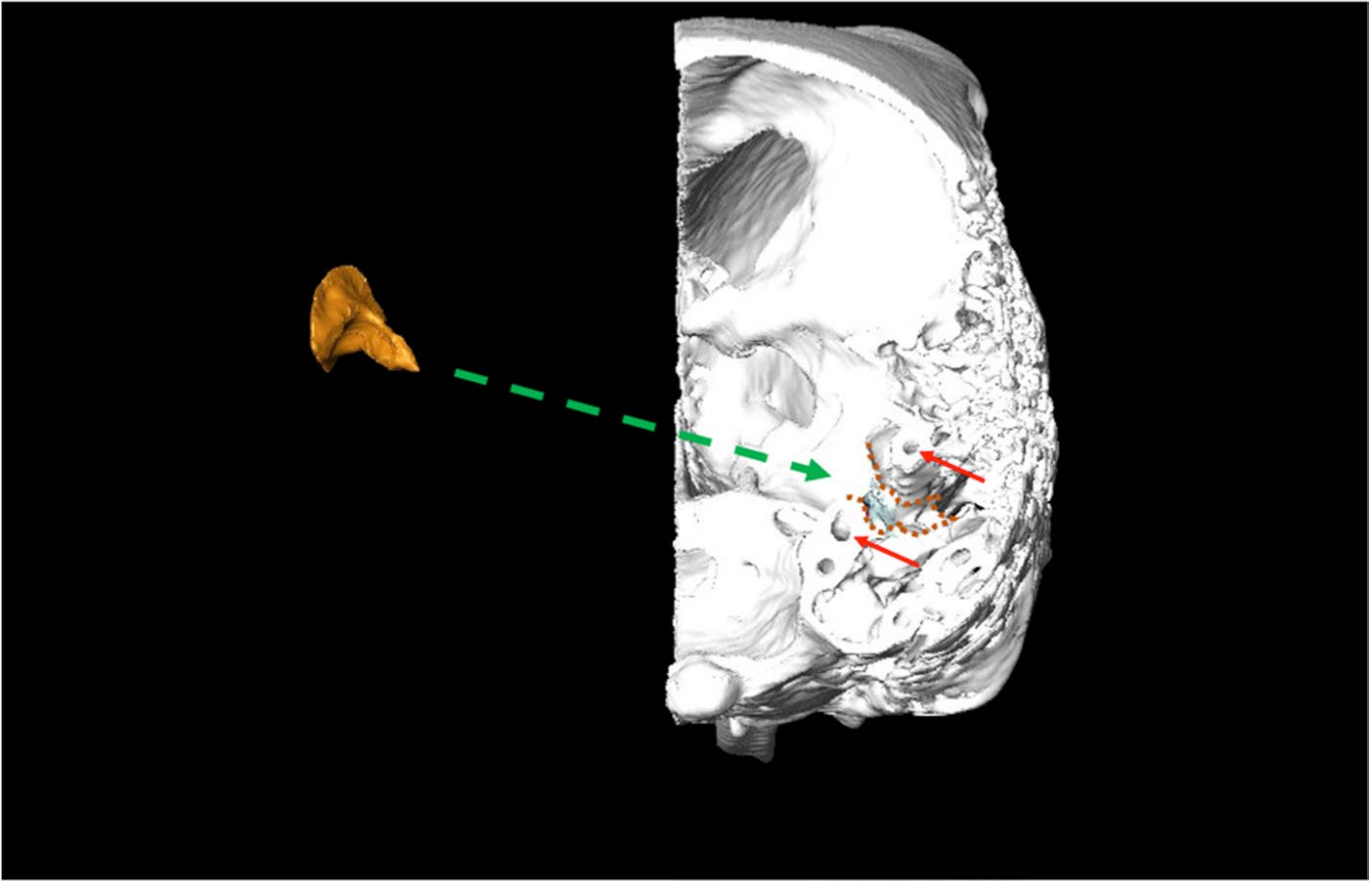
Coronal cut-out of the 3D reconstruction of the right half of *Columba livia* skull (in posterior view (left side removed). FF endocast in orange. Dashed orange lines mark the FF borders. Red arrows mark the anterior semicircular canal.

The data were processed using Amira 5.3.3 (Visualization Sciences Group, France). The FF volumes were measured twice by different users. No significant differences were detected between different measurements. This procedure was applied to our avian dataset and to both MfN and KUPRI’s (Kyoto University Primate Research Institute) CT scans (see Figs 3–6; see Supplementary Materials and Methods).

A phylogenetic tree for mammals was built based on the topology of Meredith *et al*.^70^. We used Mesquite 3.03^71^ to build and adjust the tree’s branch lengths according to divergence time between taxa. Bird phylogeny was based on Hackett *et al*.’s^72^ tree (see Supplementary Materials and Methods).

Body mass values for birds and mammals were obtained from Dunning^73^ and Smith *et al*.^74^, respectively. All values were log10 transformed.

The species were divided into ecological categories related to feeding, activity pattern, dimension of locomotion and locomotor type (see Supplementary Materials and Methods). Birds and mammals were classified according to: (1) Feeding strategy; (2) Activity pattern. Additionally, we created three more divisions for our mammalian dataset: (1) 2D/3D locomotion; (2) locomotor type; (3) agility.

We performed a log10 transformation on the original FF volume data. Total Endocast Volume minus FF (TEVr) values were then log10 transformed and included in a phylogenetically corrected regression using Mesquite 3.03^71^. The prediction intervals were mapped onto the original tip data space to detect the existence of outliers^75^ (two outliers were removed from the bird data set). Relative values were obtained by running two phylogenetic generalized least-squares regressions on FF and TEVr for mammals and birds. PGLS is a model that takes into account the phylogenetic relationships between tip data^76, 77^. We calculated phylogenetic residuals from FF vs. TEVr regressions that were used as relative FF size in the subsequent analyses. The use of phylogenetic residuals reduces variance and Type I errors in the analysis^78^. Residual normality was checked by Shapiro-Wilk and Kolmogorov-Smirnov tests and additionally with quantile-quantile plots.

Analyses of variance were performed to find out if there are significant differences in FF relative size of the created categories, on both full models and models resulting from stepwise variable removal. We used ape, nlme, car and MASS packages in R software to perform all the calculations^79^. Multiple regressions were performed using a PGLS with phylogenetic trees with divergence time (million years) as branch lengths. In the case of the mammalian analyses the tree was not ultrametric due to the presence of a fossil specimen. We tested different models of trait evolution (see Supplementary Materials and Methods).

## Electronic supplementary material

Supplementary information I

Supplementary information II

Supplementary information III

Supplementary Materials and Methods
