# Supplementary material for: Floccular fossa size is not a reliable proxy of ecology and behaviour in vertebrates
Source: Sci Rep. 2017 May 17;7:2005. doi: 10.1038/s41598-017-01981-0 (PMC5435717; doi:10.1038/s41598-017-01981-0)
Supplement: Supplementary file 4 — Supplementary Materials and Methods [file 41598_2017_1981_MOESM4_ESM.pdf]

Supplementary Materials and Methods

Floccular fossa size is not a reliable proxy of ecology and behaviour in vertebrates

Ferreira-Cardoso, S.*; Araújo, R.; Martins, N.E.; Martins, G.G.; Walsh, S.; Martins, R.M.S.; Kardjilov, N.; Manke, I.; Hilger, A. and Castanhinha, R.*

*corresponding authors – Sérgio Ferreira-Cardoso (sff.cardoso@campus.fct.unl.pt) and Rui Castanhinha (rcastanhinha@gmail.com)

X-ray micro-computed tomography (µCT) experiments

X-ray µCT scanning of mammal skulls was performed at the Helmholtz Zentrum Berlin with a micro-focus 150 keV Hamamatsu X-ray source with a tungsten target and a flat panel detector C7942 (120x120 mm, 2240×2368 pixels, pixel size 50 µm). All specimens were scanned with an acceleration voltage of 100 keV and a beam current of 95 µA, with an exposure time of 0.5 seconds since these parameters provided the best results. Image noise was reduced by using a 3-fold frame integration. The source-object distance varied between 150-220 mm and the source-detector distance was 300 mm and thus pixel size varied between 24.55-36 µm. The number of acquired projections varied between 800 and 1000. In the X-ray cabinet the sample was rotated on a precision rotation stage from Huber, Germany. We used Octupus V8.6 software to implement the back-projection algorithm with convolution and correction for cone beam, thus reconstructing the bone structure. The reconstructed 3D datasets were later down-sampled by 2x2x2 average binning using the FIJI software[1] to allow faster processing and handling.

Additional data sources

Fifteen skull CT scans were downloaded from the Kyoto University Primate Research Institute's (KUPRI) online collection (http://dmm.pri.kyoto-u.ac.jp/) see table in supplementary materials III). Although the voxel size of these scans was larger (by one order of magnitude), it did not compromise our analysis because the resolution allowed detection and a detailed segmentation of the FF.

Braincase cavity and FF volumes for *Monodelphis domestica*, *Didelphis virginiana*, *Phascolarctos cinereus*, *Dasyurus hallucatus* and *Dromiciops gliroides* were obtained from Macrini et al.[2]. We used Castanhinha et al.[3] values for *Niassodon mfumukasi* and Macrini et al.[4] for *Ornithorhynchus anatinus*.

Body mass for mammals and birds were taken from Smith et al.[5] and Dunning[6], respectively. The majority of the museum specimens that were scanned had no information about gender. During specimen selection, we only used adults, however, precise body mass of the museum specimens was also not possible to retrieve.

3D data processing and measuring

Processing consisted of five steps: 1) reorientation of the scan to obtain digital endocasts in orthogonal anatomical orientation by using the Transform Editor of Amira V5.3 and applying the transformation using a standard interpolation in extended mode while preserving voxel size; 2) semi-automatic segmentation of skull bone by using the 3D Display and Masking tool of the Segmentation Editor; 3) semi-automatic segmentation of the braincase cavity with Magic Wand tool (Brush tool was also used for slices containing foramina); 4) selection of both FF volumes by cutting using the 3D "lasso" tool - this process consisted of three further steps: a) make a sagittal cut-off of the skull to expose the periotic region; b) select the volume inside fossae adjoining the remaining endocast volume; c) cut the volume exceeding the limit (corresponding to the anterior semicircular canal) that results from the change of angle between the braincase lateral wall (periotic) and the fossa itself (see Figures 3 & 4 of the manuscript); 5) measurement of brain cavity and FF volumes (combined volume of left and right structures).

Phylogenetic trees

Divergence data were collected from the following publications: Spoor et al.[7], mainly for higher taxonomic levels; Meredith et al.[8] for Marsupialia, Arnason et al.[9] and Poux et al.[10] for Afrotheria, Nyakatura & Bininda-Emonds[11] for Carnivora, Agnarsson et al.[12] for Chiroptera,

Steppan et al.[13] and Blanga-Kanfi et al.[14] for Rodentia, and Perelman et al.[15] for Primates, to adjust branch lengths at a family taxonomic level. *Niassodon mfumukasi*[3] was added as outgroup to all the other clades and the divergence time between Anomodontia and Theriodontia (the clade in which class Mammalia is included) was fixed at 261 million years. The most primitive anomodonts were found in Dashankou locality in China[16]. There are no theriodonts in Dashankou and, therefore, we assume that divergence happened before the Lower *Pristerognathus* zone. Given that no dating is available, we consider Rubidge et al.[17] U-Pb dating of 261 million years as a minimum age for divergence. We used Hackett et al.[18] phylogenetic tree for the avian data set. Different tree topologies or branch lengths might retrieve different results. In both mammals and birds, two different tree branch transformations were applied (OU alpha and Grafen rho). Additional analyses were run using phylogenetic trees with equal branch lengths (=1). We also run analyses with trees based on Hedges et al[19] and we retrieved similar results. A discussion concerning the effects of phylogenetic uncertainty can be found elsewhere[20]. Nevertheless, we rerun our analysis with branch lengths = 1 and the results are consistent.

Ecological traits

1. Feeding strategy - (0) gatherer. (1) occasional predator. (2) predator - in which gatherers do not engage in any kind of predation. Occasional predators predate but are predominantly omnivores and predators that obtain most of their resources by hunting;
2. Activity pattern – (0) nocturnal. (1) nocturnal/diurnal. (2) diurnal - nocturnal/diurnal being the category for those animals that do not fit a strictly nocturnal or diurnal pattern.

Additionally, we created three more divisions for our mammalian data set:

1. Dimension of locomotion – (0) 2D, (1) 3D - in which groups include animals that move mainly on a horizontal plane and which consistently move both horizontally and vertically;

2. Locomotor type – (0) fossorial, (1) semiaquatic, (2) terrestrial, (3) scansorial, (4) arboreal, (5) flyer – adapted and modified from Van Valkenburgh[21], fossorials forage and shelter underground, semiaquatics forage on water but shelter on dry land or built platforms, terrestrials forage and shelter on the ground and rarely or never climb, scansorials move on the ground but regularly climb, arboreals forage and shelter on trees;

3. Agility – (0) slow, (1) medium slow, (2) medium, (3) medium fast, fast (4) – adapted from Spoor et al.[7].

The behavioral categories of the extinct *Niassodon mfumukasi* were attributed according to what have been inferred previously[3,22].

Statistical analysis

We checked for multicollinearity issues between predictors using VIF (Variance Inflation Factor)[23]. We used three R packages, namely: car, MASS and nnet. In the case of mammals, a large VIF was detected for locomotion dimension and locomotor type (see table 1). Therefore, as an internal control we ran multiple regressions, with and without these predictors, to test if they were influencing our model results. Significance was not altered.

All calculations were performed in R software[24], using the following packages: ape, nlme, MASS and car. A non-phylogenetic model and 4 models of trait evolution (Brownian Motion, OU and Grafen) were used to test the hypotheses (see tables 2 & 4). We performed stepwise regressions with bidirectional variable elimination to obtain the most parsimonious model. Stepwise regressions have advantages compared to a typical GLS because the choice of

predictive variables is automatically made taking into account the AIC (Akaike Information Criterion). AIC values were used to determine the best fitting model (table 3 & 5).

Table 1 - Generalized Variance Inflation Factors (GVIF) table showing large values (>>10) of Locomotor type and 2D/3D predictors. Values were normal when Locomotor type was removed.

	GVIF (all variables)	GVIF (-1 variable)
**Body mass**	2.82	2.23
**Agility**	7.94	3.01
**Locomotor type**	156.14	Removed
**2D/3D Locomotion**	14.06	1.40
**Feeding strategy**	4.56	2.75
**Activity pattern**	4.64	2.39

Table 2 - Results of the ANOVA (Type II) of the mammal data set. Statistics of the effect of each predictor on FF relative size variation (Chi-square value, number of degrees of freedom and p value; α = 0.05). Four models of trait evolution (non-phylogenetic, Brownian Motion, Ornstein-Uhlenbeck and Grafen) were tested. Statistics of analysis with equal branch lengths (BL=1) is shown for the best fitting model. The highlighted columns contain the statistics for the most parsimonious models (stepwise regression). These results show no significant effects of the predictive variables on FF size for the best fitting models (Brownian Motion and Grafen). OU alpha = 0.99; Grafen rho = 0.49.

	GLS	GLS – stepwise regression	GLS Brownian Motion	GLS Brownian Motion – stepwise regression	GLS Ornstein Uhlenbeck	GLS Ornstein Uhlenbeck – stepwise regression	GLS Grafen	GLS Grafen – stepwise regression	GLS Brownian Motion – BL = 1	GLS Brownian Motion – BL = 1 – Stepwise regression
**Body mass**	Chisq = 0.19; df = 1; p = 0.66	Removed	Chisq = 0.78; df = 1; p = 0.38	Removed	Chisq = 0.42; df = 1; p = 0.52	Chisq = 3.97; df = 1; p = 0.05	Chisq = 0.87; df = 1; p = 0.35	Removed	Chisq = 1.57; df = 1; p = 0.21	Removed
**Agility**	Chisq = 2.91; df = 4; p = 0.57	Removed	Chisq = 2.28; df = 4; p = 0.68	Removed	Chisq = 2.28; df = 4; p = 0.68	Removed	Chisq = 5.45; df = 4; p = 0.24	Removed	Chisq = 6.28; df = 4; p = 0.17	Removed
**Locomotor type**	Chisq = 9.35; df = 5; p = 0.10	Chisq = 10.63; df = 5; p = 0.06	Chisq = 3.63; df = 5; p = 0.60	Removed	Chisq = 7.98; df = 5; p = 0.16	Removed	Chisq = 8.64; df = 5; p = 0.12	Removed	Chisq = 9.52; df = 5; p = 0.09	Removed
**2D/3D Locomotion**	Chisq = 3.91; df = 1; p =0.05	Chisq = 5.60; df = 1; p = 0.02	Chisq = 1.44; df = 1; p = 0.23	Removed	Chisq = 3.52; df = 1; p = 0.06	Chisq = 5.02; df = 1; p = 0.02	Chisq = 0.28; df = 1; p = 0.60	Chisq = 3.06; df = 1; p = 0.08	Chisq = 0.28; df = 1; p = 0.60	Chisq = 2.44; df = 1; p = 0.12
**Feeding strategy**	Chisq = 2.64; df = 2; p = 0.28	Chisq = 4.44; df = 2; p = 0.11	Chisq = 1.85; df = 2; p = 0.40	Removed	Chisq = 6.19; df = 2; p = 0.05	Chisq = 20.24; df =2; p<0.001	Chisq = 2.19; df = 2; p = 0.33	Removed	Chisq = 2.02; df = 2; p = 0.36	Removed
**Activity pattern**	Chisq = 5.27; df = 2; p = 0.07	Chisq = 5.87; df = 2; p = 0.05	Chisq = 2.68; df = 2; p = 0.26	Removed	Chisq = 2.06; df = 2; p = 0.36	Removed	Chisq = 2.57; df = 2; p = 0.24	Removed	Chisq = 4.18; df = 2; p = 0.12	Removed

Table 3 – Comparison between models, for mammal data set, using the Akaike Information Criterion (the best fitting model is shaded). In the fourth column there is a formula (heading) by which is possible to compare AIC values between models. All models are less than 0.01 times as probable as the best fitting model to minimize the information loss, except for GLS Grafen and BL=1 stepwise regression. This clearly shows that the Brownian Motion and Grafen models are, by far, the best models for our data.

	Model	AIC	$e^{(AICmin-AICi)/2}$
**GLS**	All variables included	47.86	<0.01
	Stepwise regression	43.43	<0.01
**GLS Brownian Motion**	All variables included	42.83	<0.01
	Stepwise regression	27.98	-
**GLS Ornstein-Uhlenbeck**	All variables included	52.42	<0.01
	Stepwise regression	43.67	<0.01
**GLS Grafen**	All variables included	42.03	<0.01
	Stepwise regression	30.33	0.38
**GLS Brownian Motion BL = 1**	All variables included	41.34	<0.01
	Stepwise regression	32.21	0.12

Table 4 - Results of the ANOVA (Type II) of the bird data set. Statistics of the effect of each predictor on FF relative size variation (Chi-square value, degrees of freedom and p value; α = 0.05). 4 models of trait evolution (non-phylogenetic, Brownian Motion, Ornstein-Uhlenbeck and Grafen) were tested. Statistics of analysis with equal branch lengths (BL=1) is shown for the best fitting model. Highlighted columns contain the statistics for the most parsimonious model (stepwise regression). These results indicate a consistent significant effect of activity pattern on FF size variation. OU alpha = 0.01; Grafen rho = 0.03.

	**GLS**	**GLS – stepwise regression**	**GLS Brownian Motion**	**GLS Brownian Motion – stepwise regression**	**GLS Ornstein Uhlenbeck**	**GLS Ornstein Uhlenbeck – stepwise regression**	**GLS Grafen**	**GLS Grafen – stepwise regression**	**GLS Brownian Motion – BL = 1**	**GLS Brownian Motion – BL = 1 – Stepwise regression**
**Body mass**	Chisq = 2.29; df= 1; p= 0.13	Chisq = 2.20; df= 1; p= 0.14	Chisq = 2.27; df =1; p= 0.13	Chisq = 2.33; df= 1; p= 0.13	Chisq = 2.28; df= 1; p= 0.13	Chisq = 2.55; df= 1; p= 0.11	Chisq = 2.25; df= 1; p= 0.13	Removed	Chisq = 1.64; df= 1; p= 0.20	Removed
**2D/3D Locomotion**	Chisq <0.01; df= 1; p= 0.98	Removed	Chisq = 0.01; df= 1; p= 0.93	Removed	Chisq = 0.00(0); df= 1; p= 0.99	Removed	Chisq <0.01; df= 1; p= 0.98	Removed	Chisq = 0.01; df= 1; p= 0.92	Removed
**Feeding**	Chisq = 2.91; df= 2; p= 0.23	Removed	Chisq = 10.92; d= 2; p<0.01	Chisq = 11.59; df= 2; p<0.01	Chisq = 6.57; df= 2; p= 0.04	Chisq = 7.47; df= 2; p= 0.02	Chisq = 2.88; df= 2; p= 0.24	Removed	Chisq = 8.83; df= 2; p= 0.01	Chisq = 8.79; df= 2; p= 0.01
**Activity pattern**	Chisq = 7.10; df= 1; p= 0.01	Chisq = 7.96; df= 1; p <0.01	Chisq = 10.99; df= 1; p<0.01	Chisq = 11.31; df= 1; p<0.01	Chisq = 8.77; df= 1; p<0.01	Chisq = 9.10; df= 1; p<0.01	Chisq = 7.09; df= 1; p= 0.01	Chisq = 7.10; df= 1; p= 0.01	Chisq = 10.64; df= 1; p< 0.01	Chisq = 10.49; df= 1; p< 0.01

Table 5 – Comparison between models, for bird data set using the Akaike Information Criterion (AIC). The best fitting model is shaded. In the 4th column there is a formula (heading) by which is possible to compare AIC values between models. Brownian Motion trait evolution models are the best fitting, with Ornstein-Uhlenbeck and BL=1 being those with the least fit, which means a stepwise regression Brownian Motion GLS is the best model to explain FF size variation.

	Model	AIC	$e^{(\mathrm{AICmin}-\mathrm{AICi})/2}$
**GLS**	All variable included	-22.56	0.04
	Stepwise regression	-24.69	0.13
**GLS Brownian Motion**	All variables included	-26.79	0.37
	Stepwise regression	-28.78	-
**GLS Ornstein-Uhlenbeck**	All variables included	-20.56	0.02
	Stepwise regression	-22.69	0.05
**GLS Grafen**	All variables included	-20.56	0.02
	Stepwise regression	-23.09	0.06
**GLS Brownian Motion BL = 1**	All variables included	-13.34	<0.01
	Stepwise regression	-15.53	<0.01

Supplementary materials and methods bibliography

1. Schindelin, J. *et al.* Fiji: an open-source platform for biological-image analysis. *Nat Meth* **9,** 676–682 (2012).
2. Macrini, T. E., Muizon, C. D., Cifelli, R. L. & Rowe, T. Digital cranial endocast of Pucadelphys andinus, a Paleocene metatherian. *Journal of Vertebrate Paleontology* **27,** 99–107 (2007).
3. Castanhinha, R. *et al.* Bringing Dicynodonts Back to Life: Paleobiology and Anatomy of a New Emydopoid Genus from the Upper Permian of Mozambique. *PLOS ONE* **8,** e80974 (2013).
4. Macrini, T. E., Rowe, T. & Archer, M. Description of a cranial endocast from a fossil platypus, Obdurodon dicksoni (Monotremata, Ornithorhynchidae), and the relevance of endocranial characters to monotreme monophyly. *J. Morphol.* **267,** 1000–1015 (2006).
5. Smith, F. A. *et al.* Body Mass of Late Quaternary Mammals. *Ecology* **84,** 3403–3403 (2003).
6. Dunning, J. B. *CRC handbook of avian body masses*. (Taylor & Francis, 2008).
7. Spoor, F. *et al.* The primate semicircular canal system and locomotion. *PNAS* **104,** 10808–10812 (2007).
8. Meredith, R. W., Westerman, M., Case, J. A. & Springer, M. S. A Phylogeny and Timescale for Marsupial Evolution Based on Sequences for Five Nuclear Genes. *J Mammal Evol* **15,** 1–36 (2007).
9. Arnason, U. *et al.* Mitogenomic relationships of placental mammals and molecular estimates of their divergences. *Gene* **421,** 37–51 (2008).
10. Poux, C., Madsen, O., Glos, J., de Jong, W. W. & Vences, M. Molecular phylogeny and divergence times of Malagasy tenrecs: Influence of data partitioning and taxon sampling on dating analyses. *BMC Evolutionary Biology* **8,** 102 (2008).
11. Nyakatura, K. & Bininda-Emonds, O. R. Updating the evolutionary history of Carnivora (Mammalia): a new species-level supertree complete with divergence time estimates. *BMC Biology* **10,** 12 (2012).

12. Agnarsson, I., Zambrana-Torrelio, C. M., Flores-Saldana, N. P. & May-Collado, L. J. A time-calibrated species-level phylogeny of bats (Chiroptera, Mammalia). *PLoS Curr* **3,** (2011).
13. Steppan, S. J., Storz, B. L. & Hoffmann, R. S. Nuclear DNA phylogeny of the squirrels (Mammalia: Rodentia) and the evolution of arboreality from c-myc and RAG1. *Molecular Phylogenetics and Evolution* **30,** 703–719 (2004).
14. Blanga-Kanfi, S. *et al.* Rodent phylogeny revised: analysis of six nuclear genes from all major rodent clades. *BMC Evolutionary Biology* **9,** 71 (2009).
15. Perelman, P. *et al.* A Molecular Phylogeny of Living Primates. *PLOS Genet* **7,** e1001342 (2011).
16. Liu, J., Rubidge, B. & Li, J. A new specimen of Biseridens qilianicus indicates its phylogenetic position as the most basal anomodont. *Proceedings of the Royal Society of London B: Biological Sciences* rspb20090883 (2009). doi:10.1098/rspb.2009.0883
17. Rubidge, B. S., Erwin, D. H., Ramezani, J., Bowring, S. A. & Klerk, W. J. de. High-precision temporal calibration of Late Permian vertebrate biostratigraphy: U-Pb zircon constraints from the Karoo Supergroup, South Africa. *Geology* **41,** 363–366 (2013).
18. Hackett, S. J. *et al.* A phylogenomic study of birds reveals their evolutionary history. *science* **320,** 1763–1768 (2008).
19. Hedges, S. B., Marin, J., Suleski, M., Paymer, M. & Kumar, S. Tree of Life Reveals Clock-Like Speciation and Diversification. *Mol Biol Evol* msv037 (2015). doi:10.1093/molbev/msv037
20. Villemereuil, P. de, Wells, J. A., Edwards, R. D. & Blomberg, S. P. Bayesian models for comparative analysis integrating phylogenetic uncertainty. *BMC Evolutionary Biology* **12,** 102 (2012).
21. Van Valkenburgh, B. Locomotor diversity within past and present guilds of large predatory mammals. *Paleobiology* **11,** 406–428 (1985).
22. Laaß, M. Virtual reconstruction and description of the cranial endocast of Pristerodon mackayi (Therapsida, Anomodontia). *Journal of Morphology* **276,** 1089–1099 (2015).

23. Mundry, R. in *Modern Phylogenetic Comparative Methods and Their Application in Evolutionary Biology* (ed. Garamszegi, L. Z.) 131–153 (Springer Berlin Heidelberg, 2014). doi:10.1007/978-3-662-43550-2_6

24. Team, R. C. *R: A language and environment for statistical computing. R Foundation for Statistical Computing, Vienna, Austria. 2013*. (2014).
